# An immunocompetent, multifluorescent resource for real-time analysis of osteosarcoma progression, metastasis, and response to therapy

**DOI:** 10.1126/sciadv.aed6938

**Published:** 2026-10-02

**Authors:** Taiana C. Leite, Jue Hou, Xuehui Geng, Luigi Mancinelli, Abigail Sedlacek, Devanshi A. Nayak, Karen E. Schoedel, Kurt R. Weiss, Robert J. Binder, Charles P. Lin, Giuseppe Intini

**Affiliations:** ^1^ Department of Oral and Craniofacial Sciences, University of Pittsburgh School of Dental Medicine, Pittsburgh, PA, USA.; ^2^ Department of Periodontics and Preventive Dentistry, University of Pittsburgh School of Dental Medicine, Pittsburgh, PA, USA.; ^3^ Center for Craniofacial Regeneration, University of Pittsburgh School of Dental Medicine, Pittsburgh, PA, USA.; ^4^ Center for Systems Biology and Wellman Center for Photomedicine, Massachusetts General Hospital, Harvard Medical School, Boston, MA, USA.; ^5^ Department of Immunology, University of Pittsburgh School of Medicine, Pittsburgh, PA, USA.; ^6^ Department of Pathology, University of Pittsburgh School of Medicine, Pittsburgh, PA, USA.; ^7^ Hillman Cancer Center, University of Pittsburgh Medical Center, Pittsburgh, PA, USA.; ^8^ Department of Orthopaedic Surgery, University of Pittsburgh Medical Center, Pittsburgh, PA, USA.; ^9^ Pittsburgh Center for Interdisciplinary Bone and Mineral Research, University of Pittsburgh, Pittsburgh, PA, USA.; ^10^ Department of Medicine, Division of Hematology and Oncology, University of Pittsburgh School of Medicine, Pittsburgh, PA, USA.

## Abstract

Despite substantial treatment improvements, the 5-year survival rate for metastatic osteosarcoma (OS) is still as low as 20%. Immunocompetent genetic mouse models of OS have been introduced, enabling in vivo OS studies. Here, we present the ROCCIA mouse, a quintuple-mutant extension of these models that develops multifluorescent OS tumors, allowing distinct fluorescently labeled OS cell populations to be identified and traced both within tumors and in the circulation. In addition, this model enables postnatal inactivation of the *p53* and *Rb1* genes, thus resembling development and progression of OS in humans. Using this model, we show that osterix (*Osx*)–expressing fluorescent tumor cells appear in circulation upon OS development and predominate in lung metastases, thus representing a potential target for future therapies. We also show that the immunological landscape of this model’s tumor microenvironment mimics the previously described human OS microenvironment. Therefore, this immunocompetent, quintuple mutant, multifluorescent mouse model represents a powerful resource to analyze OS tumor development and metastasis in real time and to test translational therapies.

## INTRODUCTION

Osteosarcoma (OS) is the most common primary bone malignancy and the second leading cause of cancer-related deaths in children and young adults (*1*–*3*). Most OS deaths are due to metastases to the lungs, which typically develop in ∼30% of patients (*4*, *5*). With the advent of chemotherapy, survival rates for OS have improved substantially. However, only around 20% of patients with metastatic OS survive after 5 years—an alarming figure that has not changed in the past 40 years (*1*). An important obstacle to improving OS prognosis is the absence of an immunocompetent animal model that mimics human disease, demonstrates spontaneous lung metastases (LM), and facilitates the discovery of the cellular dynamics and immune processes that modulate metastatic OS biology (*6*–*8*).

Animal models, including canine and murine, have been crucial for understanding OS biology, genetics, and LM, ultimately aiding in treatment development and improving survival (*6*, *9*). While canine models offer a high natural incidence of OS (20 times higher than humans), they present limitations such as age differences (geriatric dogs) and variable heritability (*10*, *11*). Consequently, OS research has relied on murine models involving the transplantation of mouse- or human-derived cell lines or tumor fragments into immunodeficient animals (*9*). However, these models have important drawbacks: Long-used cell lines accumulate genetic changes, compromising their reliability for studying OS biology and treatment response (*6*, *9*). Furthermore, using established OS cells, whether from cell lines or patient donors in immunocompromised mice, precludes studying the actual tumorigenesis and metastasis processes, including progressive malignant transformation, immune editing, stepwise mutation acquisition (*6*, *12*), and tumor microenvironment (TME) significance (*13*).

Fortunately, over a decade ago, researchers developed transgenic mouse models of OS that, by means of Cre recombinase–mediated conditional inactivation of *p53* and *Rb1* or activation of Notch signaling in cells of the osteoblastic lineage, can spontaneously develop OS lesions in an immunocompetent environment (*14*–*19*). This pioneering work has provided immunocompetent mouse models that mimic OS formation in vivo. However, they still showed limitations. For instance, all these mouse models rely on the constitutive inactivation or activation of genes during embryogenesis. Consequently, the neoplastic process is initiated in embryo, therefore not recapitulating the human neoplastic process, which occurs upon somatic mutations, postnatally. In these mouse models, the development of a large proportion of non–OS/soft tissue tumors is also reported [as high as 59% (*14*, *15*)] in parallel to the development of OS. In addition, the anatomical distribution of OS lesions does not always mirror human disease (i.e., a high incidence of lesions affecting the craniofacial bones) (*14*–*18*).

Another topic of interest in addressing the issue of metastatic spread in cancer is circulating tumor cells (CTCs). One of the major barriers to OS research is represented by the inability to trace these cells, which are responsible for the development of LM. This is of paramount importance in developing a cure for cancer in general and for OS in particular, given that, as mentioned, metastatic disease represents the main cause of death among patients with OS (*1*–*3*). CTCs are extremely rare, with an estimated frequency of 1 to 100 CTCs per 107 white blood cells (*20*), and most sorting methods of CTCs rely on cell size– and antibody-based microfluidic devices (*20*). However, such devices are not as effective in isolating mesenchymal CTCs, especially OS, because of lack of specific surface markers (*21*). Therefore, an animal model that provides the opportunity to trace, isolate, and characterize CTCs would contribute substantially to the development of therapeutic tools that specifically target circulating and metastasizing OS cells.

Last, cancer immunotherapy, specifically immune checkpoint blockade (ICB), is now a first-line option for many patients with cancer, in part because of the higher-than-historical-objective clinical responses and high tumor specificity. However, ICB has not offered benefit to patients with OS (*22*). A prerequisite for successful ICB is the preexistence of tumor-infiltrating T cells, which have been shown to be lacking in OS (*23*–*25*). Having an immunocompetent mouse model of OS that effectively resembles the human disease is essential for establishing attributes of successful immunotherapy.

Here, we report on the development of a multitransgenic mouse model of postnatal OS that is immunocompetent and multifluorescent, improving upon the previously developed models. We named this mouse model ROCCIA, in memory of a child who lost his life to OS. To generate this mouse model, we used three different fluorochromes expressed under the control of three different osteoblastic lineage–specific promoters: (i) the *Prx1*/*Prrx1* promoter/enhancer to control the expression of enhanced green fluorescent protein (EGFP), (ii) the *Osx*/*Sp7* promoter to control the expression of mCherry, and (iii) the *Col1* promoter to control the expression of cyan fluorescent protein (CFP). A tamoxifen-inducible Cre recombinase (CreER) under the control of the *Prx1*/*Prrx1* promoter/enhancer (Prx1/Prrx1-CreER-EGFP) was used to inactivate *p53* and *Rb1* genes postnatally in osteoprogenitor cells that express EGFP. Here, first, we show that the postnatal OS tumors of ROCCIA mice mirror tumors observed in the OS human diseases. Then, by means of the multifluorescent proteins embedded in the ROCCIA bone cells, we study how OS cells at various stages of osteoblastic differentiation populate the OS primary and metastatic lesions and the circulation, identifying osterix (*Osx*)–expressing cells as the tumor cells present in LM and in the circulation. Substantiating the importance of this observation, we show that the immunological landscape of the TME of the ROCCIA mouse is similar to the previously described human OS TME, including being devoid of T cell infiltrates.

## RESULTS

### Generation of the ROCCIA mouse and development of OS tumors

To generate the ROCCIA mouse model, first, we generated a transgenic multifluorescent mouse, with fluorochrome expression driven by three different promoters that identify three different stages of differentiation of the osteoblastic lineage (Fig. 1A). The *Prx1* promoter was used to induce the expression of EGFP in skeletal stem/progenitor cells (*26*), the *Osx/Sp7* promoter was used to induce the expression of mCherry protein in osteoprogenitor cells (*27*), and the *Col1a1* promoter was used to induce the expression of CFP in osteoblastic precursors and mature osteoblasts (Fig. 1B) (*28*, *29*). First, we crossed mice of the C57BL/6 background with mice carrying the Prx1-CreER-EGFP transgene (*26*) to obtain offspring expressing the EGFP and the tamoxifen-inducible CreER in skeletal stem/progenitor cells. Subsequently, we generated a Prx1-CreER-EGFP^+/+^;Osx-mCherry^+/+^;Col1-CFP^+/+^ mouse by crossing with mice carrying the Osx-mCherry (*27*) and Col1-CFP (*28*, *29*) transgenes. Then, to generate the ROCCIA mouse, we used a *Prx1*-driven tumor suppressor gene inactivation strategy previously described (*15*). However, whereas the latter relied on the constitutive *Prx1*-driven inactivation of the *p53* and *Rb1* tumor suppressors, we used tamoxifen-inducible *Prx1*-driven inactivation (CreER), thus inactivating the *p53* and *Rb1* genes postnatally, in *Prx1*-expressing skeletal stem/progenitor cells. Specifically, the Prx1-CreER-EGFP^+/+^;Osx-mCherry^+/+^;Col1-CFP^+/+^ mice were crossed into a mouse line of the C57/Bl6 background bearing floxed alleles of *Rb1* and *p53* to obtain a congenic colony of Prx1-CreER-EGFP^+/+^;Osx-mCherry^+/+^;Col1-CFP^+/+^;p53^lox/lox^;Rb1^lox/lox^ mice that, upon treatment with tamoxifen, exhibit inactivation of *p53* and *Rb1* (Fig. 1C).

**Fig. 1.**
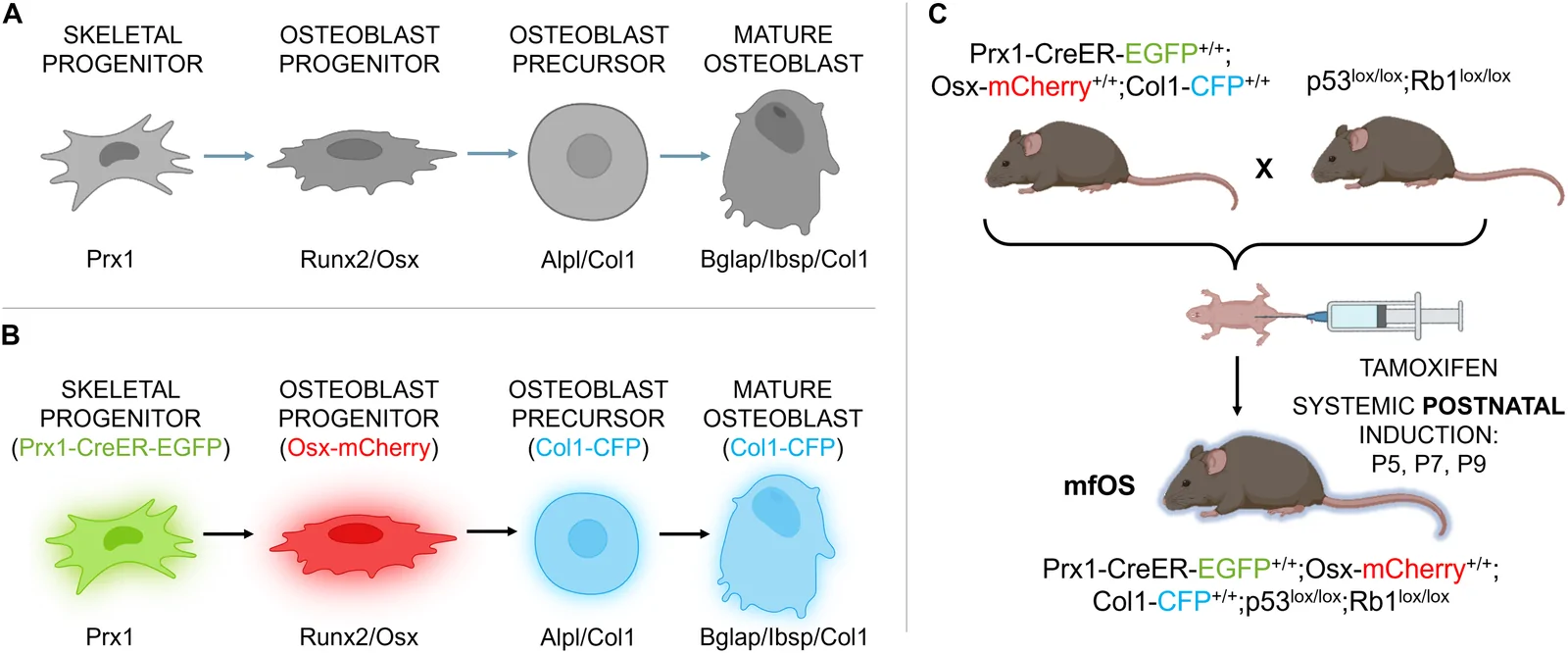
Generation of the ROCCIA mouse. (**A**) Working model for different stages of osteoblast differentiation. During embryogenesis and postnatally, skeletal progenitors express *Prx1* (*27*, *28*). These cells differentiate into osteoblast progenitors expressing *Runx2*, which controls the expression of *Osx*. Upon induction by *Runx2* and *Osx*, osteoblast progenitors start expressing alkaline phosphatase (*Alpl*) and *Col1*, becoming osteoblast precursors (*29*). Osteoblast precursors become mature osteoblasts when expressing osteocalcin (*Bglap*) and bone sialoproteins (*Ibsp*), along with *Col1* (*30*). Created in BioRender. Campos Leite, T. (2026) https://BioRender.com/d8gvvqx. (**B**) Multifluorescent osteoblastic differentiation. By crossing a Prx1-CreER-GFP mouse with Osx-mCherry and Col1-CFP transgenic mice, a multifluorescent osteoblastic stage–specific mouse model can be generated, whereby skeletal progenitors are green-fluorescent, osteoblast progenitors are red-fluorescent, and osteoblast precursors and mature osteoblasts are cyan-fluorescent. Created in BioRender. Campos Leite, T. (2026) https://BioRender.com/fdj4jat. (**C**) Development of a multifluorescent mouse model of OS (ROCCIA mouse). By crossing the multifluorescent mouse model of osteoblastic differentiation with a mouse carrying floxed alleles of the *p53* and the *Rb1* genes, upon postnatal injection of tamoxifen (at P5, P7, and P9), the inactivation of *p53* and *Rb1* is induced in postnatal *Prx1*-expressing cells. Created in BioRender. Campos Leite, T. (2026) https://BioRender.com/mpv6oqu. Created in BioRender. Campos Leite, T. (2026) https://BioRender.com/n6lutq4.

To evaluate the ability of the ROCCIA mice to develop multifluorescent OS, we injected a total of 35 mice with tamoxifen (intraperitoneally, 0.05 mg/g) at postnatal day 5 (P5), P7, and P9 to inactivate *p53* and *Rb1* in postnatal *Prx1*-expressing skeletal stem/progenitor cells (Fig. 2A). Starting at week 22 of age, mice were monitored biweekly, radiographically, and clinically (Fig. 2A). We started to detect initial tumor lesions radiographically at ∼29 weeks of age (latency period), with metastases usually developing ∼4 weeks later (Fig. 2A). The average age for euthanasia was 36.6 weeks (Fig. 2A). A total of 32 of 35 mice developed tumors (penetrance of 91.4%) (Fig. 2B), with similar sex distribution (13 of 14 males and 19 of 21 females developed tumors). Twelve of 32 tumor mice (37.5%) developed internal organ metastases, while 27 of 32 mice (84.3%) presented multiple lesions in other skeletal segments (Fig. 2B).

**Fig. 2.**
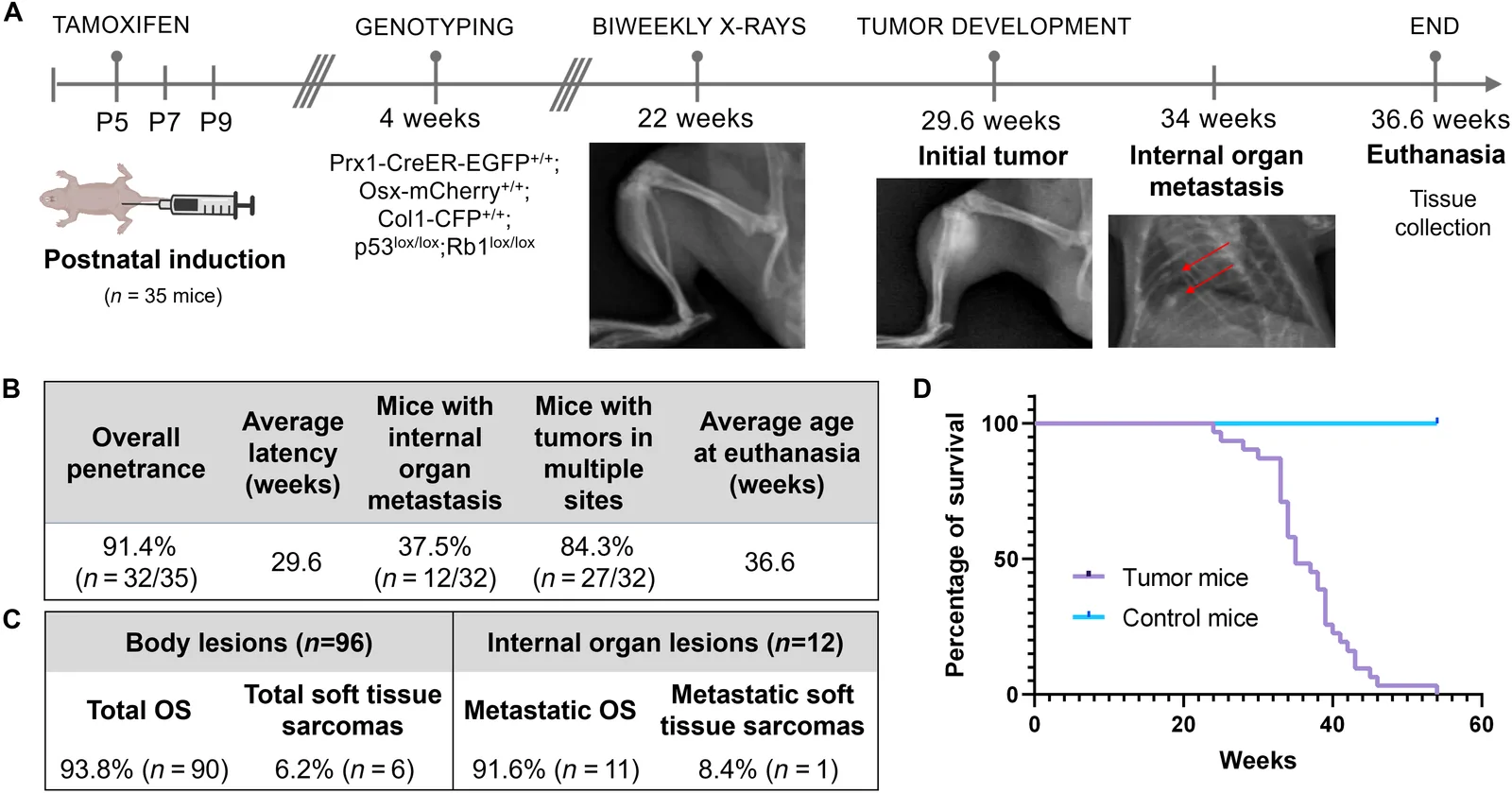
ROCCIA model and tumor induction. (**A**) Postnatal tumor detection and induction timeline. Pups received three injections of tamoxifen at P5, P7, and P9. At 4 weeks of age, the genotype of each animal was confirmed with PCR. At 22 weeks, biweekly radiographs were initiated, and at around 30 weeks, initial signs of OS tumors appeared. At around 34 weeks, internal organs started to develop metastases (red arrows), and at around 37 weeks, they were euthanized for experimental purposes. Created in BioRender. Campos Leite, T. (2026) https://BioRender.com/65ihnuh. (**B**) Summary data of the development of OS lesions in the ROCCIA mouse colony. (**C**) Distribution of tumor type and location. (**D**) Kaplan-Meier survival curve comparing tumor mice (*n* = 31) to negative control mice (*n* = 10). All control mice were still alive at 54 weeks (end point).

Histological analyses confirmed that the vast majority (93.8%) of all tumors in bones/limbs were OS, while only six (6.2%) were soft tissue sarcomas (Fig. 2C). Eleven metastases to internal organs were OS (91.6%), and only one (8.4%) was soft tissue sarcoma (Fig. 2C). Four animals presented two or more initial tumors simultaneously. Two of these had an initial soft tissue sarcoma together with initial OS in separate sites. Only three mice had a single initial soft tissue sarcoma, while the other 28 mice presented initial OS lesions.

We injected 10 mice not carrying the Prx1-CreER-EGFP transgene (Prx1-CreER-EGFP^−/−^;Osx-mCherry^+/+^;Col1-CFP^+/+^;p53^lox/lox^;Rb1^lox/lox^) with tamoxifen and used them as tumor-negative controls, monitoring them throughout their life span in a similar way to the tumor mice. While all test/tumor mice required euthanasia by 37 weeks of age (Fig. 2D), at 54 weeks of age, all control mice survived and showed no signs of tumor development nor change in behavior.

### Development of OS in the ROCCIA mice mirrors the development of OS in humans

Most initial tumors were detected in femurs and/or tibias (74%; Fig. 3A, left); subsequent tumor formations were also mostly detected in femurs and/or tibia (66%; Fig. 3A, right). Radiographic images reliably detected the presence of OS lesions in long bones and in other bones (for instance, calvarial bones) and presented with a mixture of radiopaque and radiolucent areas (Fig. 3, B and C).

**Fig. 3.**
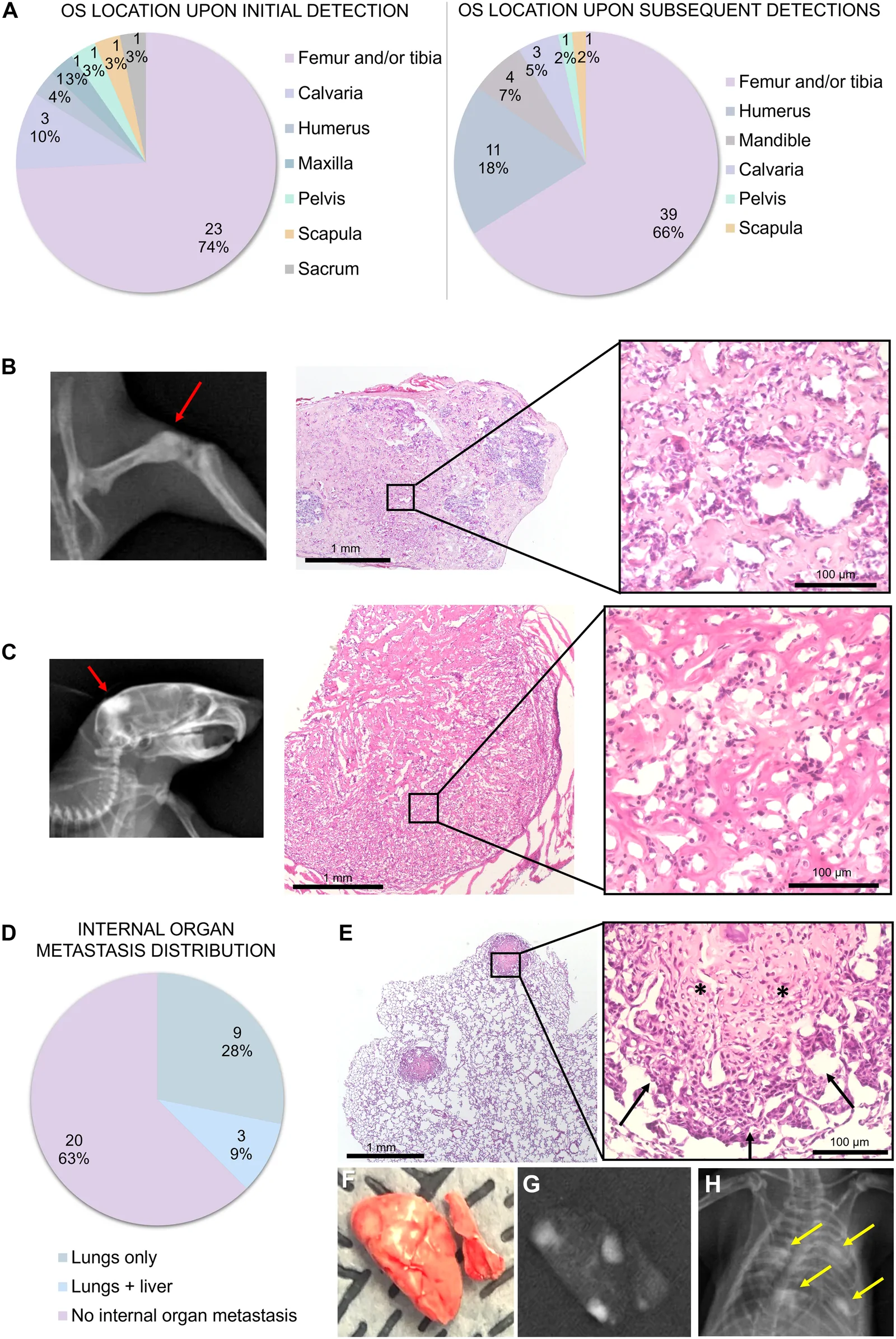
OS development and metastatic spread in the ROCCIA mouse resemble human disease. (**A**) Bone distribution of detected OS lesions. (**B** and **C**) Representative radiographic (left; red arrows) and histological (right; HE, 40× and 400× inset) images of OS lesions in femur (B) and calvarium (C). (**D**) Internal organ distribution of detected OS metastases. (**E** to **H**) Representative images of OS metastases to lungs. (E) Histological image (HE, 40× and 400× inset) showing two nodules with central osteoid (asterisks) and a more cellular periphery at the interface of the pulmonary parenchyma (black arrows). (F) Gross image of metastatic nodules in dissected lungs and corresponding radiographic image (G). (H) Radiographic image of thorax showing radiopaque metastatic nodules in lungs (yellow arrows).

Independent from the location, histologically, tumors demonstrated a consistent morphology of typical OS (Fig. 3, B and C), with the presence of variable amount of osteoid interconnecting immature bone intimately associated with tumor cells, showing a range of nuclear pleomorphism, coarse chromatin, nuclear contour irregularities, mitotic activity, and necrosis. Tumor cells trapped in the osteoid matrix were occasionally “normalized,” exhibiting less cytologic atypia. The osteoid matrix was either partially mineralized or unmineralized, often a mixture of both. There was morphologic tumor heterogeneity evidenced by tumor areas devoid of osteoid matrix; these areas exhibited increased numbers of tumor giant cells (Fig. 3, B and C).

All metastatic tumors to internal organs were detected in lungs or in lungs and liver simultaneously (Fig. 3D). Histological images of the metastases also confirmed a typical OS morphology with the presence of osteoid in variable abundance (Fig. 3E). Nodules were evident in gross evaluation of the surface of the lungs and were occasionally radiopaque (Fig. 3, F to H). As OS LM are typically seen on the pleura or pleural reflections, this spatial pattern also recapitulates the human disease.

### OS lesions of the ROCCIA mice present distinct fluorescent profiles

To characterize the fluorescent expression of the skeletal OS lesions in the ROCCIA mice, we performed intravital microscopy on bone tumors using an imaging approach previously used in our laboratory (*30*). Given that intravital microscopy works better on flat bone (*31*, *32*), we performed this analysis on tumor lesions that developed in the calvaria. All analyzed tumors (*n* = 9 mice and 14 tumors) showed expression of at least one of three fluorochromes, with the presence of variable numbers of mCherry-positive cells (red), EGFP-positive cells (green), and CFP-positive cells (blue) in each tumor (Fig. 4A). Specifically, in 100% of the primary OS lesions, cells expressing mCherry alone were detected, whereas cells coexpressing mCherry and CFP were detected in 79% of the primary OS lesions. In addition, cells expressing GFP alone or CFP alone were detected in 43 or 79% of the lesions, respectively (Fig. 4A).

**Fig. 4.**
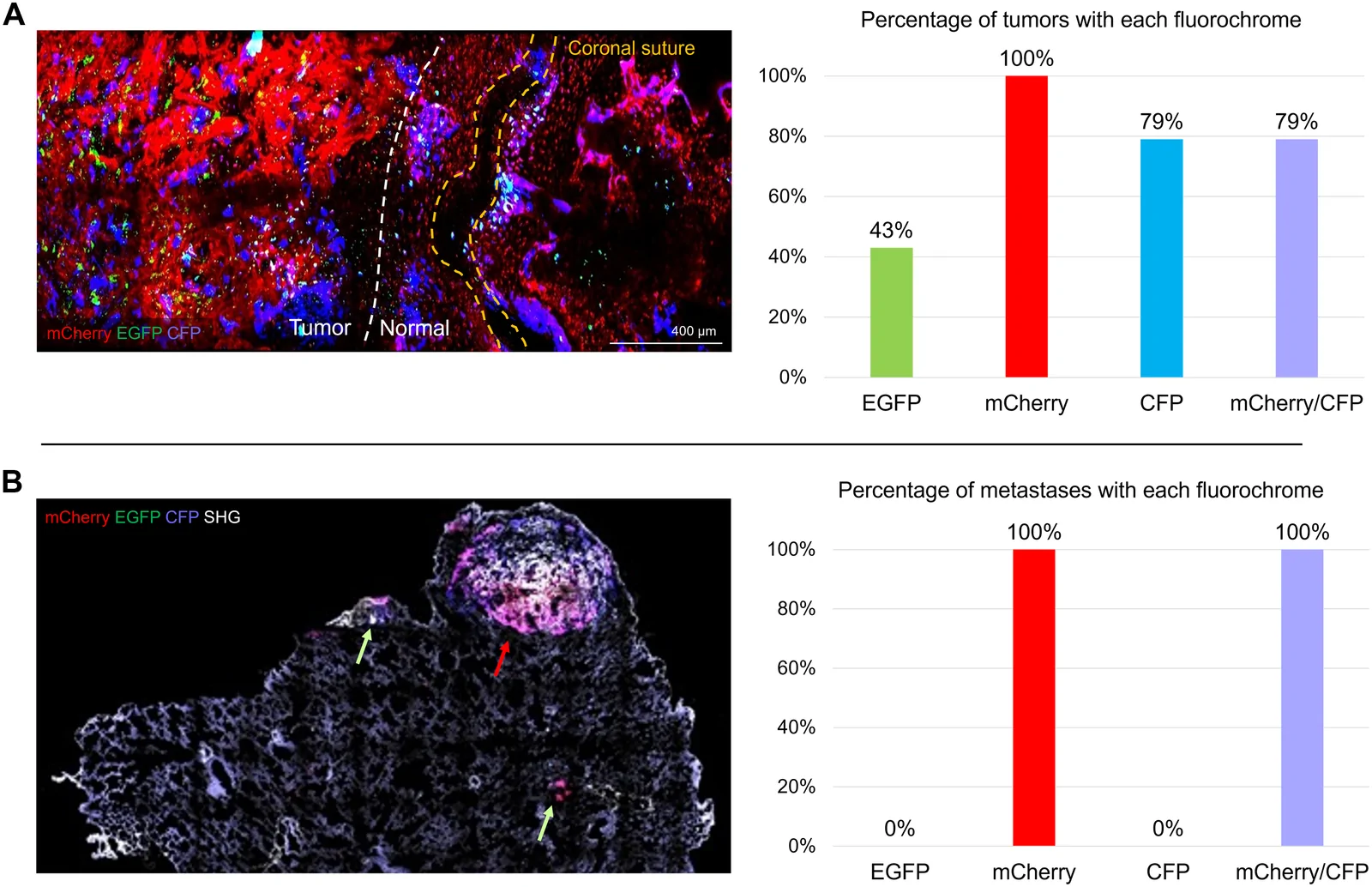
Fluorescence expression of ROCCIA mouse tissues shows prevalence of *Osx*-expressing cells. (**A**) Intravital imaging of a calvarial tumor. On the left, the representative image shows stronger signals on the tumor compared to the normal bone for all three fluorochromes—EGFP, mCherry, and CFP. On the right, fluorochrome expression distribution in bone OS as per intravital imaging is shown, displayed as the percentage of tumors containing cells expressing a certain fluorochrome (EGFP, mCherry, or CFP) or a fluorochrome combination (mCherry/CFP). (**B**) Ex vivo imaging of a metastatic lung. On the left, the representative image shows a metastatic nodule (red arrow) and small metastatic foci (green arrows). On the right, fluorochrome expression distribution in metastatic OS to internal organs as per ex vivo imaging is shown, displayed as the percentage of metastases containing cells expressing a certain fluorochrome (EGFP, mCherry, or CFP) or a fluorochrome combination (mCherry/CFP).

Given that internal organs are not anatomically accessible for intravital microscopy, we performed confocal fluorescence imaging on ex vivo histological sections of metastatic lesions to lungs and livers (*n* = 3 mice; three LM and two liver metastases). All metastases contained only cells expressing mCherry alone or coexpressing mCherry and CFP (Fig. 4B).

### In vivo and ex vivo flow cytometry identifies the prevalence of *Osx* (mCherry)–expressing cells among the CTCs

The presence of cells expressing mCherry in 100% of the metastases differs from the more heterogeneous distribution of fluorochromes that we observed in primary bone tumors, suggesting that mCherry-expressing cells may be responsible for metastases. To test this hypothesis, we performed in vivo flow cytometry (IVFC) for noninvasive monitoring of multifluorescent CTC (Fig. 5A) (*33*). Mice were imaged for 30 s, with 10 imaging sessions for each animal. In ROCCIA mice bearing no tumors, fluorescent cells were not detected (Fig. 5B), while in ROCCIA mice bearing tumors, fluorescent CTCs were identified (Fig. 5, C to E). Within all the fluorescent cells identified, mCherry-expressing cells were more prevalent than others, with more than 60% expressing only the mCherry fluorochrome and more than 20% coexpressing CFP (Fig. 5F). Supporting the IVFC data, additional quantitative studies using traditional ex vivo fluorescence-activated cell sorting showed that ∼40% of all nucleated circulating cells expressed mCherry (Fig. 5G). This confirms that mCherry is the dominant fluorochrome expressed in circulation.

**Fig. 5.**
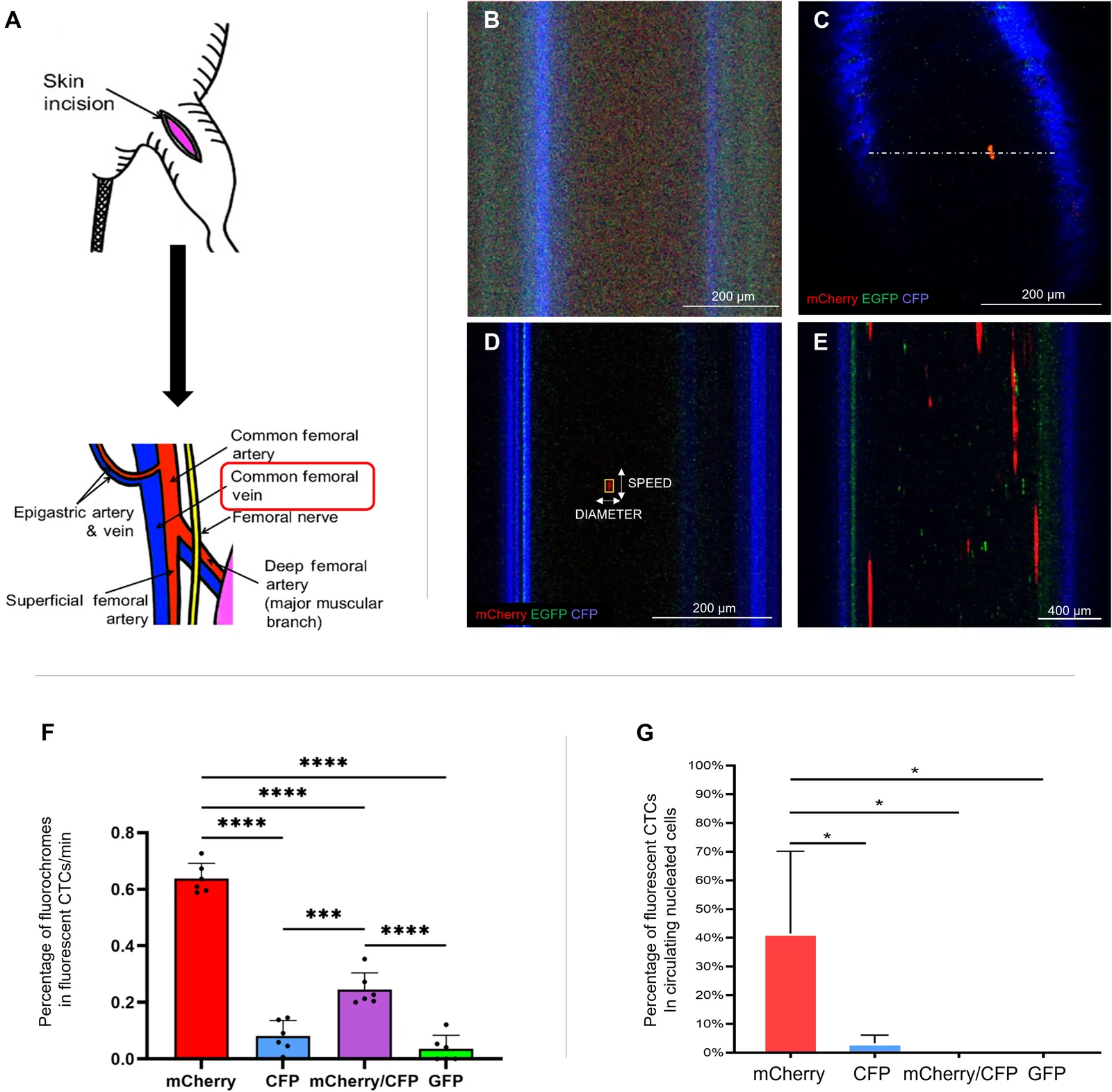
Fluorescence expression of ROCCIA mouse CTCs shows the prevalence of *Osx*-expressing cells. (**A**) Schematics of IVFC. A skin incision exposes the common femoral vein to be imaged. (**B** to **E**) Representative images of IVFC. (B) Imaging of a healthy control animal in 1D scan for mCherry, EGFP, and CFP. (C) Imaging of a ROCCIA mouse in 2D scan. (D) Generation of masks for each CTC in a 1D scan image. Each CTC was fitted into the smallest box containing the cell, and the cell diameter is defined as the width of the box and the cell speed is inversely proportional to the length of the box and total CTCs captured in 30 s. (E) Total CTCs captured in 30 s in 2D scan. (**F**) Percentage of fluorescent CTCs captured per minute during tumor progression for each cell type (*n* = 6 mice) detected through IVFC. (**G**) Fluorescent CTCs detected by means of ex vivo flow cytometry in circulating nucleated cells (*n* = 3 mice with metastatic OS). Data are presented as the means ± SD. *P < 0.05, ***P < 0.0001, and ****P < 0.00001 as determined by ANOVA and Dunnet’s post hoc tests.

### The immunological landscape of tumors in ROCCIA mice phenocopies human OS

We previously analyzed and immunophenotyped a cohort of 145 sarcomas (including OS) freshly isolated from patients (*25*) and documented the range of variability in patient tumors with respect to immune infiltrates, the number of tumor mutations and neoepitopes, and antigenicity. We examined the immunological landscape of primary and metastatic tumors from ROCCIA mice to compare to OS and to establish the baseline in immune cell frequency. To evaluate immune responses to tumors in ROCCIA mice, we harvested primary OS tumors (identified by radiography) from mouse femurs at week 36. Tumors were digested and stained for total immune cells (CD45+) and major immune subsets to determine the frequencies associated with tumor samples. Data from four ROCCIA tumors phenocopied human OS in the following ways: (i) Two ROCCIA mice (T75 and T85) had tumors that were heavily associated with immune cells (CD45+), and two mice (T84 and T89) had tumors with very few immune cells (Fig. 6). The range of immune cells in OS from ROCCIA mice phenocopies what we have observed in human OS (*25*). (ii) All tumors had skewing of the macrophage (Mac) population to the M2 type, which is characterized by a suppressive or protumor phenotype (*34*). We did not observe any M1 Macs, which typically contribute to antitumor immunity. The other notable population was monocytes/granulocytes, which include myeloid-derived suppressor cells that are immunosuppressive like M2 Macs. These findings are similar to an independent large-scale analysis of human OS where Macs in general, but particularly M2 Macs, are dominant (*35*). (iii) Regardless of some range in frequencies, antitumor immune cells, generally comprising effector CD4+ and CD8+ T cells, natural killer cells (NK cells), and dendritic cells, were a small fraction of immune cells associated with the tumor. These data provide an exclusive first look at the immune profile of tumors in ROCCIA mice.

**Fig. 6.**
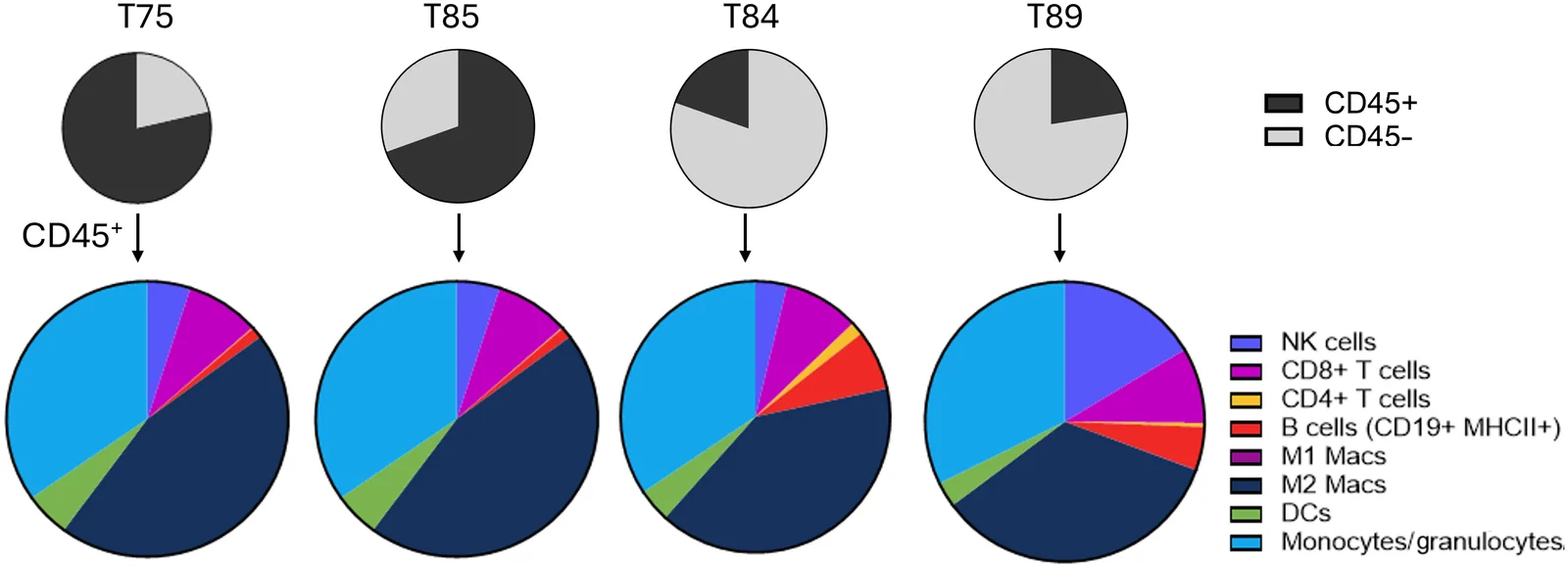
Profile of immune cell infiltrates in primary OS of ROCCIA mice. Tumors were harvested from four ROCCIA mice (T75, T84, T85, and T89) at week 36 and enzymatically digested into single cells. Cells were stained with cell surface markers for major immune cell types. The top row shows the frequency of CD45+ cells in tumor. The bottom row shows the subset analysis of CD45+ cells. In each sample, a small percentage of CD45+ cells was not identified with markers for major immune cells.

We also examined spleen and lymph nodes from ROCCIA mice because changes in these tissues sometimes reflect immunological reactivity to tumors (Fig. 7). T84 and T89 had unusual spleens with a large number of CD45− cells and exhibited splenomegaly. Within the few CD45+ cells obtained from these large spleens, the CD8+ T cell population was significantly expanded. T75 and T85 mice had relatively normal spleens in terms of CD45+ frequency and size. These mice had an expanded CD8+ T cell population, but other major immune cell frequencies appeared normal. These data indicate that there is a CD8+ T cell response to tumors, and given that the tumors themselves are devoid of these effector cells, tumor-mediated mechanisms may be preventing infiltration. The splenomegaly and low number of CD45+ cells in spleen and tumor-draining lymph nodes of T84 and T89, on the other hand, suggest tumor cell penetration of lymphatic and blood circulation (Fig. 7). This is a precursor step to establishing metastatic lesions in more distant tissues, including LM. Analysis of blood is typically used as a clinical surrogate of immune reactivity to tumors (*36*, *37*), so we examined the blood of the four ROCCIA mice (Fig. 8). T84 and T89 had more CD45− cells than CD45+ cells. Incidentally, these were the mice with no LM, suggesting that sheer increased circulating tumor burden does not necessarily predict LM. Significant expansion of CD4+ and CD8+ T cells was observed, and the overall frequencies of immune cells mirrored what was observed in lymph nodes and spleen.

**Fig. 7.**
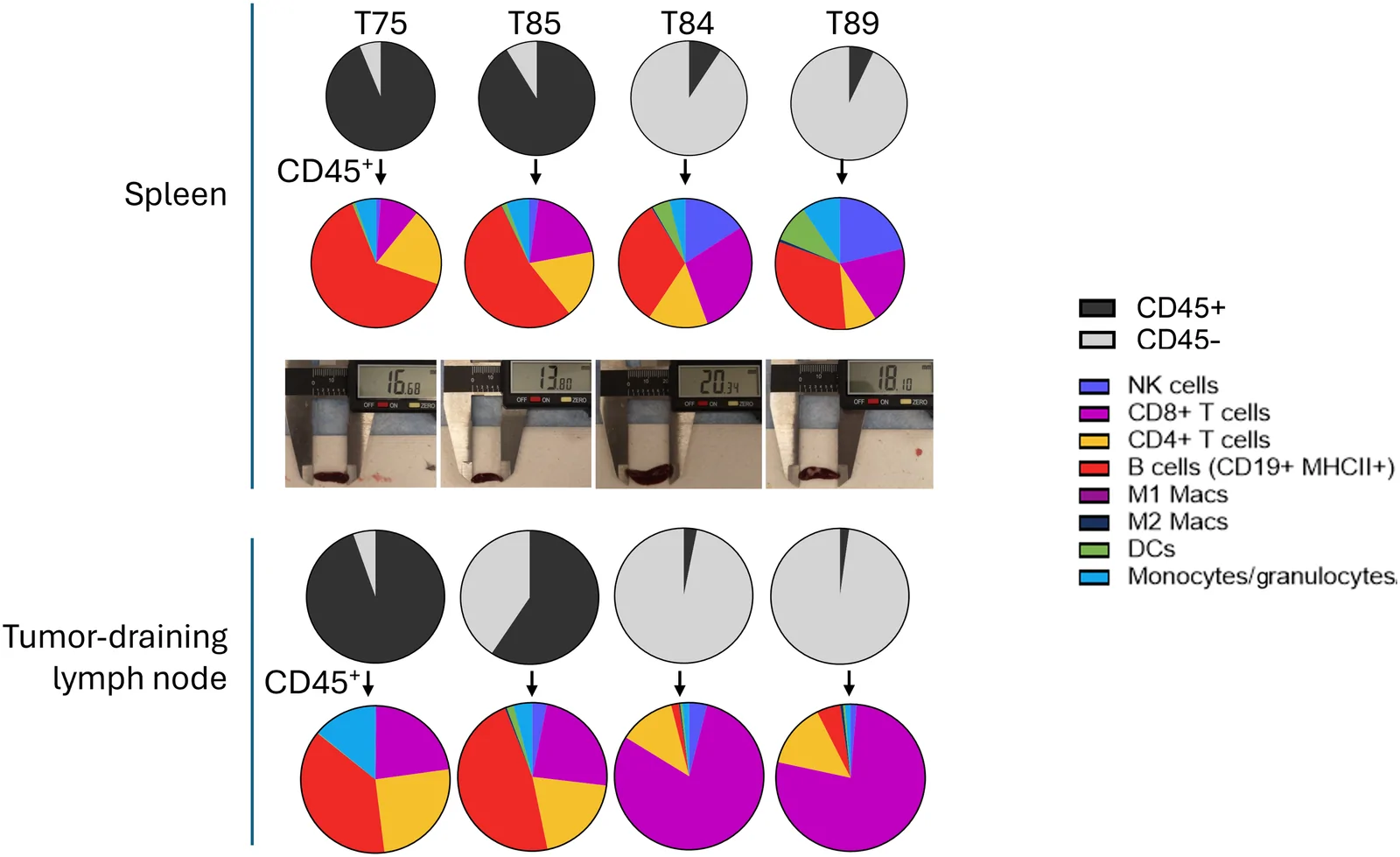
Profile of spleens and tumor-draining lymph nodes from ROCCIA mice. Spleens and tumor-draining lymph nodes were harvested from four ROCCIA mice (T75, T84, T85, and T89) at week 36 and processed into single cells. Cells were stained with cell surface markers for major immune cell types. Top rows for each tissue show the frequency of CD45+ cells in tumor. Bottom rows show the subset analysis of CD45+ cells. In each sample, a small percentage of CD45+ cells was not identified with markers for major immune cells. The size of the spleen (mm) is shown.

**Fig. 8.**
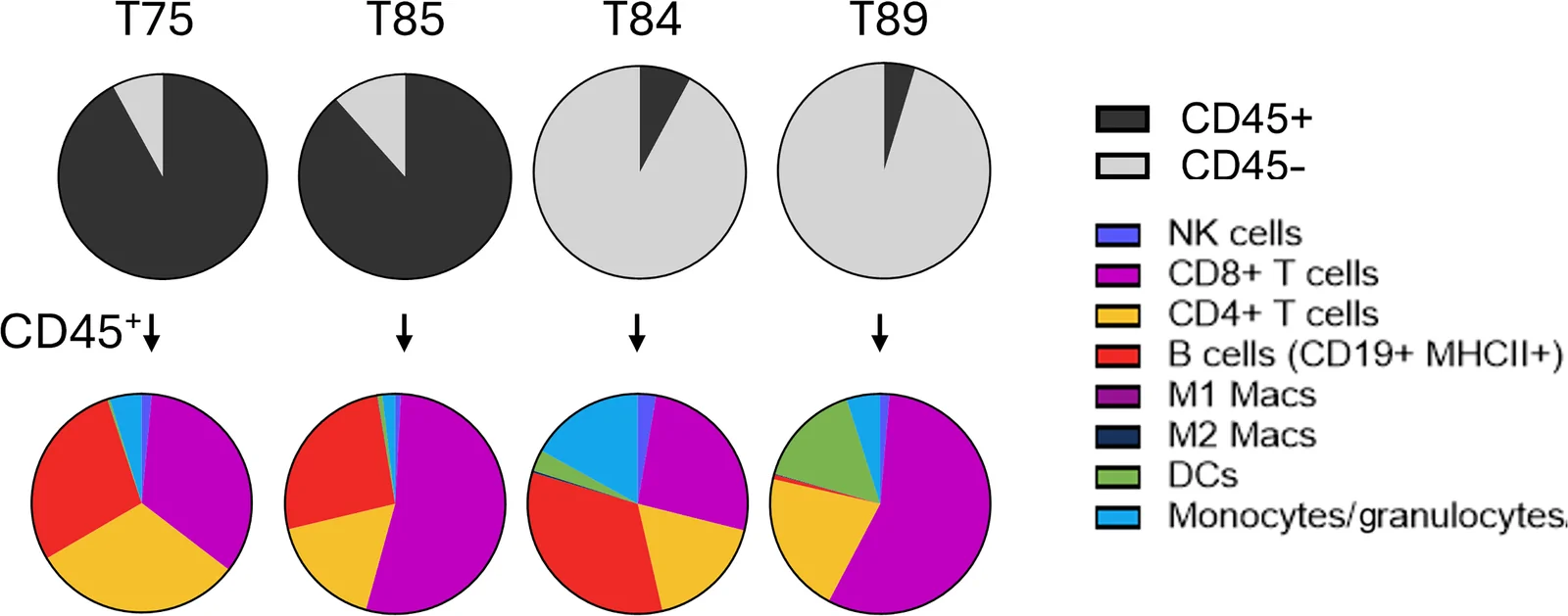
Profile of immune cell infiltrates in the blood of ROCCIA mice. Blood was harvested from four ROCCIA mice (T75, T84, T85, and T89) at week 36 and processed into single cells. Cells were stained with cell surface markers for major immune cell types. The top row shows the frequency of CD45+ cells in tumor. The bottom row shows the subset analysis of CD45+ cells. In each sample, a small percentage of CD45+ cells was not identified with markers for major immune cells.

## DISCUSSION

We developed the ROCCIA mouse with the purpose of establishing a more reliable animal model to study OS and identifying the cell population responsible for the development of LM. By means of three fluorescent transgenes, whose expression is guided by three different promoters during different stages of osteoblastogenesis, we detected various fluorescent cells within the primary lesion, in the metastases, and in the bloodstream. In addition, by using an inducible Cre-recombinase system, we inactivated the *p53* and *Rb1* tumor suppressor genes postnatally, thus proving the opportunity to mimic the development of human OS.

The reported results indicate that, in comparison to other OS mouse models that rely on the inactivation of *p53* and *Rb1* (*15*–*17*), the ROCCIA mouse is the transgenic OS mouse model that most closely resembles human development and the clinical behavior of the disease. In contrast to previously reported genetic models, the ROCCIA mouse resembles the human condition the most, presenting a more similar location distribution and a higher LM prevalence. In other mouse models, OS lesions develop most frequently in craniofacial bones (jaw, calvaria, and snout) (*16*, *17*), whereas the ROCCIA mouse develops lesions mainly in long bones. Given the postnatal inactivation within the skeletal stem/progenitor cell population, we also selectively induced the development of OS primary lesions almost exclusively in the bone tissues and observed a very low occurrence of other types of tumors. In comparison, a high prevalence of soft tissue sarcomas was observed when the *Prx1* deleter was used to inactivate *p53* and *Rb1* during embryogenesis, and only 24% of the mice presenting OS lesions produced LM (*15*).

The observed penetrance of OS in ROCCIA mice is not 100%, given that 32 of 35 mice developed tumors. We believe that two factors may be contributing to this result. First, we recognize that cancer immunosurveillance can, occasionally, be successful. Cancer immunosurveillance has been well documented by us (*38*, *39*) and other labs (*40*, *41*) in many mouse models. In systems of tumor induction, by genetically engineered mice (*41*) or with carcinogens (*38*–*40*), there are often mice that do not develop tumors. This is actually a promising and expected observation in the ROCCIA mice, which have an intact immune system, one we will pursue in future studies. Second, it should also be considered that Cre recombination is often incomplete; specifically, per our previous experience, the Prx1-CreER-EGFP construct used in this work typically achieves a 90 to 95% efficiency (*30*). While secondary, this aspect may have contributed to a penetrance of less than 100%.

The latency period of the ROCCIA model is ∼29 to 30 weeks and, therefore, within the first quarter of murine life. This also resembles the age of occurrence of the human OS, which peaks in the first two decades of life (*42*, *43*). With respect to location, the most common sites of human OS are metaphyseal regions of long bones, especially distal femur/proximal tibia (∼75%) and proximal humerus (∼11%), although OS may develop in other bones, such as jaws and pelvis (*44*–*46*). Similarly, the OS lesions in the ROCCIA model presented a very similar distribution, with 74% of primary OS lesions affecting femur and/or tibia and 10% occurring in humerus. We conclude that the ROCCIA mouse represents an advancement of the previously generated mouse models of OS, in that it better resembles the human condition, with a significantly higher number of lesions that originate in the bone tissues, a higher percentage of OS cases that metastasize to the lungs, and a nearly identical preference to femur and tibia.

The expression of the three fluorochromes under the control of the three different promoters allowed us to identify different osteoblastic-like cells within the total cells of the OS lesions. Given that *Prx1* is expressed in progenitor cells (*30*, *47*), while *Osx* and *Col1* are expressed in more differentiated cells of the osteoblastic lineage (*27*–*29*, *48*, *49*), we had initially expected the *Prx1*-expressing (EGFP+) cells, as the most undifferentiated ones, to represent the most abundant cell population within the OS lesions. However, unexpectedly, they were very rare, and the most abundant fluorochrome was mCherry, expressed by *Osx*-expressing cells. Moreover, according to our results, the mCherry-expressing cells are likely the ones leading to LM, as they are the most abundant CTCs, as shown in the IVFC experiments. Further validating this result, a previous study did not report the Prx1-CreER-EGFP transgene to be expressed in lungs and livers (*26*), allowing us to conclude that the metastatic lesions originate from cells expressing *Prx1* and their descendants. The expression of *Osx*, however, appears to induce a phenotype more compatible with spreading, favoring circulation and extraskeletal colonization. Undoubtedly, this observation warrants further investigation to fully characterize the Osx-expressing cells and evaluate their capacity to drive metastasis.

At the histopathological level, the OS that developed in the ROCCIA mice also morphologically resembled the human OS, both in the primary and metastatic lesions. Lesions presented with malignant cells surrounded by a ubiquitous presence of osteoid. Tumors showed heterogeneity, often showing areas of cartilage and fibrous tissue in addition to the osteoid (*42*, *45*).

The OS lesions of the ROCCIA mice also present optimal qualities in terms of immune profiling. While traditional murine models of OS based on cell transplantation in immunomodulated mice provide some insight into the disease, such models have major deficiencies. First, they lack de novo establishment of an accurate TME and therefore lack the onset of immunosurveillance mechanisms, vasculature, tumor immune editing, and innervation. Second, the transplantable and xenograft models consist of a relatively genetically invariable and homogeneous tumor cell population implanted in syngeneic (e.g., BALB/c) mice with no variation in immune response, tumor antigens, or ICB response. Genetic variability in humans and the heterogeneous OS tumor and TME are not reflected in these murine models. Third, immunotherapy and, specifically, administration of anti-PD1 and anti-CTLA have shown some benefit toward rejection of K7M2 transplantable tumors (*50*), a clear departure from a lack of such response in patients with OS (*22*). Allowing for the long-term evolution of a developing tumor in an environment of constant immunological pressure is important for tumor editing and antigenicity (*25*, *51*). Given the short progression time, these transplantable models are thus impractical to fully understand the immunobiology of OS in a longitudinal manner. In contrast, our immunological characterization of the OS lesions of the ROCCIA mice indicates that there are notable similarities between ROCCIA tumors and human OS in terms of range of immunological reactions, personalized and distinct immune responses to tumors, and suppressive TME. In addition, our data demonstrate the feasibility of analyzing immune cell populations in various tissues (e.g., primary tumor, blood, lymph node, and spleen), providing a baseline (preimmunization or therapy) for experiments where antitumor T cells will be primed with immunization regimens or chemotherapy; demonstrate an expanded T cell response in nontumor tissues; demonstrate significant CTCs in blood and secondary lymphoid structures as precursors to LM; and lastly, substantially improves on transplantable tumor models of OS that do not recapitulate human disease.

While the ROCCIA mouse demonstrates several advantages over previous OS models, it still presents limitations. One such limitation refers to the genetic alterations that give rise to the tumor—in this case, the inactivation of *p53* and *Rb1*. Mutations in these genes are present in a large number of human OS cases; however, they are not the only ones present. Consequently, our model does not represent the full heterogeneous genetic spectrum of the disease. In addition, although resembling the disease observed in humans, the long latency time for the ROCCIA mouse to develop tumors also warrants careful time management on the part of the researchers, given that these mice require hosting and maintenance in the animal facilities for a relatively long period of time. Another limitation, representing the most substantial difference between the OS lesions observed in the ROCCIA mice and in humans, is the rapid development, in most animals, of multiple OS lesions occurring in different skeletal segments within the same animal. This distribution is rarely observed in human OS, where, typically, there is an initial single OS tumor in the bone, eventually followed by LM or bone metastasis (*44*). Differently, in the ROCCIA mouse, the rapidly developing multiple bone tumors may not necessarily be secondary to a first primary lesion because inactivation of *p53* and *Rb1* occurs in all cells expressing *Prx1* throughout the body, not just in a single bone. Last, regarding the methodology used to monitor the development of OS in the ROCCIA mouse, it should be noted that radiographic monitoring, although simple and convenient, lacks the resolution to detect microlesions, neither in primary OS nor in metastatic OS, at least in live animals. Thus, the reported statistics (i.e., regarding latency) could vary if more accurate methodologies, such as in vivo micro–computed tomography, were used to detect the initial lesions.

In summary, we have developed an improved immunocompetent model of spontaneous OS that is postnatally induced and that more closely mimics human OS when compared to other OS mouse models. This model has the unique feature of bearing various fluorescent cells, which can be traced through various imaging techniques and provide insight into the types of osteoblastic OS cells participating in different steps of tumorigenesis and metastasis. By means of this unique feature, we were able to identify *Osx*-expressing cells as the cell population present in circulation and in LM, suggesting that these cells may be the cells to target in therapies that aim at preventing the development of LM. The immunological landscape of the ROCCIA mouse, being similar to the one observed in human OS, further validates these results.

We anticipate that the availability of the ROCCIA mouse as a scientific resource will be well received by investigators in the field of OS research, and we will gladly share this resource with accredited researchers. Thus, many future directions open up. Researchers may use the ROCCIA mouse to fully characterize each fluorescent population of cells and investigate their roles in OS tumor and metastasis development. Immuno-oncologists interested in the mechanisms of immunosurveillance may use this mouse to dynamically evaluate the response of fluorescent cells to immunostimulation or blockade therapies. Other investigators may directly evaluate the effects of novel drugs on the distribution and suppression of the OS fluorescent cells. We believe that the ROCCIA mouse represents a resource for future studies that focus on the development and spread of OS or explore novel therapeutics.

## MATERIALS AND METHODS

### Multifluorescent OS (ROCCIA) mouse

To develop the model, first, we generated a transgenic multifluorescent mouse, with fluorochrome expressions driven by cells in different stages of the osteoblastic lineage (Fig. 1). We crossed mice of the C57/Bl6 background to generate the Prx1-CreER-EGFP^+/+^;Osx-mCherry^+/+^;Col1-CFP^+/+^ mouse. *Prx1* drove the expression of EGFP, *Osx* drove the expression of mCherry, and *Col1* drove the expression of CFP.

Subsequently, we generated the ROCCIA mouse. To this end, we crossed the Prx1-CreER-EGFP^+/+^;Osx-mCherry^+/+^;Col1-CFP^+/+^ mice with mice of the C57/Bl6 background bearing floxed alleles of tumor suppressor genes *p53* and *Rb1*. Resulting mice were, therefore, Prx1-CreER-EGFP^+/+^;Osx-mCherry^+/+^;Col1-CFP^+/+^;p53^lox/lox^;Rb1^lox/lox^. The use of CreER allowed us to control the inactivation of *p53* and *Rb1* in terms of location—that is, exclusively in skeletal progenitor cells (expressing *Prx1*)—and time—that is, postnatally, upon tamoxifen induction of Cre recombinase, at P5, P7, and P9 (Fig. 2A). Starting at P5, we administered tamoxifen (T5648, Sigma-Aldrich, MO) in three doses, 48 hours apart, by intraperitoneal injections of 0.05 mg/g diluted in corn oil (Sigma-Aldrich, MO) using a 31G needle.

The Prx1-CreER-EGFP mice were donated by Murakami and collaborators (*26*). The Col1-CFP mice were donated by Kalajzic and collaborators (*28*, *29*). The p53- and Rb-floxed mice were obtained from the Jackson Laboratory and were previously described (*52*), as well as the Osx-mCherry mice (*27*). All animals were bred, housed, and handled at the University of Pittsburgh, Department of Laboratory Animal Resources, in accordance with the University of Pittsburgh Institutional Animal Care and Use Committee (IACUC) regulations. Specifically, cages had filter tops and were kept in racks under laminar air flow, with humidity between 45 and 60% and temperature between 67°F (19.4°C) and 69°F (20.6°C). Light/dark cycles were kept at 12:12 hours. The study was reviewed and approved by the University of Pittsburgh IACUC under protocol numbers 18114062, 20066890, 23063363, and 24105774.

Upon weaning, at around 4 weeks of age, we verified genotypes, according to standard protocol, by analyzing DNA from ear punches. Briefly, genomic DNA was extracted using the REDExtract-N-Amp Tissue PCR Kit (Sigma-Aldrich, MO), according to the manufacturer’s directions. Then, we genotyped samples using polymerase chain reaction (PCR). Master mixes were prepared with Taq 5X Master Mix (New England Biolabs, MA) and primers for the transgenes Prx1-CreER-EGP, Osx-mCherry, Col1-CFP, *p53*, and *Rb1* (Table 1); reactions were carried out in a thermal cycler using customized protocols. Last, we analyzed amplicons using gel electrophoresis (1% agarose gel, 100 V) on the basis of the length of bands. Mice presenting a negative genotype for Prx1-CreER-EGFP were used as nontumor controls.

**Table 1.** Primers used for genotyping. bp, base pairs.

Genes	Sequence 5′ → 3′	Target	Primer type
*Prx1-*CreER-EGFP	TGT TGG CAA AGG GGT TTT C	Prx1-Cre (∼400 bp)	Forward
	AGG CAA ATT TTG GTG TAC GG		Reverse
*Osx-*mCherry	ATC CTC TTG GAG TCC CTG GT	Osx-mCherry (∼450 bp)	Forward
	GCG CAT GAA CTC CTT GAT GA		Reverse
*Col1-*CFP	GGA AAG ATG TGG GAA GTG GA	Col1a1-GFP (∼290 bp)	Forward
	AAC AGC TCC TCG CCC TTG		Reverse
*p53*	GGT TAA ACC CAG CTT GAC CA	p53 (∼270 bp)	Forward
	GGA GGC AGA GAC AGT TGG AG		Reverse
*Rb1*	CTC TAG ATC CTC TCA TTC TTC CC	Rb1 (∼200 bp)	Forward
	CCT TGA CCA TAG CCC AGC AC		Reverse

Starting at 22 weeks of age, mice underwent biweekly full-body ventrodorsal and lateral radiographic monitoring (Fujinon, Soyee Product Inc., Seoul, Korea) under intraperitoneally injected ketamine hydrochloride (100 mg/kg) (Covetrus North America, OH) and xylazine (6 mg/kg) (Covetrus North America, OH) cocktail anesthesia. We also monitored mice for overall weight, appearance, and behavior. Upon detection of tumors, timing and location were recorded (Fig. 2A).

When mice showed initial signs of distress, they were euthanized through either CO_2_ asphyxiation with cervical dislocation or cardiac puncture with thoracotomy. Subsequently, they were dissected and examined for the presence of tumors. Tumors were resected and appropriately stored for further processing.

### ROCCIA mouse fluorescence imaging

We used a multiphoton microscope to obtain intravital images of the ROCCIA mouse (*n* = 9 mice and 14 bone tumors) and to perform IVFC (*n* = 6 mice). We acquired intravital images with a custom multiphoton microscope equipped with a tunable Insight X3 laser, as previously described (*53*). Briefly, the femtosecond excitation laser beam was focused through a 60× water-immersion objective (Olympus LUMFLN60XW) to excite the fluorophores. We used 20-mW power on the sample to efficiently excite the fluorophores in the animals. An area of 400 μm by 400 μm was scanned [two-dimensional (2D) scanning mode], and the fluorescence emissions and second harmonic generation (SHG) from the bone and collagen fibers were directed to photomultiplier tubes with proper dichroic mirrors for detection in the nondescanned geometry. Specifically, we used bandpass filters of 370/100 nm to detect the SHG signal, 525/50 nm for GFP and CFP signal detection, and 617/73 nm for mCherry detection. To efficiently separate CFP and GFP, we used 775- and 980-nm excitation lasers to excite CFP and GFP, respectively.

For intravital imaging, we anesthetized the animals with an induction dose of 1.5% isoflurane and secured them on a heated customized animal stage for stable data acquisition. The tumor site was then surgically exposed using a number 15 scalpel blade for en face imaging. For ex vivo imaging, upon euthanasia, histological sections of metastatic lesions to lungs and the liver of three of nine mice were analyzed using conventional fluorescence microscopy.

### ROCCIA mouse IVFC

We performed IVFC to trace CTCs in ROCCIA mice in real time. We anesthetized the animals and secured them on a heated customized animal stage for stable data acquisition with our multiphoton microscope (described above). We removed the hair on the inner side of the hindlimb, created a skin flap to expose the common femoral vein, and imaged the area directly (Fig. 5A). First, we imaged the areas with a normal 2D scanning mode to identify the femoral vein (Fig. 5B). Then, we stopped the galvo mirror to repeatedly scan through a fixed position perpendicular to the blood flow over the vessel at a rate of 15 kHz (1D scanning mode). For each session, we recorded the CTCs in the blood flow for 30 s, with 10 imaging sessions for each animal. The fluorescence signal emitted from the CTCs was detected using the same detection channel as the normal 2D scanning mode. The CFP- and mCherry-positive cells were captured with a 775-nm excitation wavelength, and GFP-positive cells were captured with 980-nm excitation.

For each CTC, the corresponding mask was fitted with the smallest box containing the area (Fig. 5D). Assuming that the CTCs travel in a straight line, the diameter of the CTC is positively correlated with the width of the box, and the flow speed of the CTC is negatively correlated with the length of the box as follows

D=C×w (1)

$$v=\frac{D}{L}\times f$$ (2)

in which *D* is the diameter of the CTC; *v* is the speed of the CTC; *w* is the width of the box in pixel number; *L* is the length of the box in pixel number; *C* is the pixel size, which is 0.4 μm in our experiment setup; and *f* is the scanning frequency of the system, which is 15 kHz.

We analyzed the IVFC data using MATLAB (The MathWorks Inc., MA). The images were thresholded to reduce the noise, and masks for mCherry-, CFP-, and GFP-positive cells were generated from the corresponding channel. The mCherry and CFP double-positive cells were defined as those with more than 50% of the pixels having both mCherry and CFP signals.

### ROCCIA mouse ex vivo flow cytometry

We collected whole blood from 34-week-old mice via cardiac puncture into heparinized tubes. Red blood cells were removed using Red Blood Cell Lysis Buffer (Roche, cat. no. 11814389001), according with the manufacturer’s specifications. Then, we performed data acquisition for nucleated cells using a BD FACSAria flow cytometer (BD Biosciences). Compensation settings were optimized before each run using BrightComp eBeads Compensation Beads (Invitrogen, cat. nos. A10514, A54742, and A54743). We collected a minimum of 20,000 single events for each sample and analyzed the data using FlowJo software (version 10.8.1, BD Life Sciences). The analysis used a sequential gating strategy to exclude debris, aggregates, and doublets, and the results were reported as the percentage of fluorescent cells within the total collected cell population. Data are presented as the mean standard error of the mean (SEM) for *n* = 3 replicates per experimental group.

### Histology

Upon dissection, tumor samples were immediately washed with phosphate-buffered saline (PBS) solution and fixed with 4% paraformaldehyde in PBS for 2 to 4 hours in ice. They were subsequently washed three times with PBS for 10 min, after which they were decalcified in 10% EDTA (pH 7.4). Subsequently, tissues were placed in a 30% sucrose solution for 24 hours, after which they were embedded in optimal cutting temperature compound (O.C.T. Tissue-Plus, Fisher Healthcare, TX) and cryosectioned in 6- to 8-μm-thick sections (Cryostat, Leica Biosystems, IL). Last, they were stained with hematoxylin and eosin (HE), as per the standard protocol (HE staining kit, Abcam, MA). Each HE glass slide was carefully examined by a medical pathologist expert in bone oncology (K.E.S.) to establish diagnosis. Additional unstained sections of LM were used for ex vivo fluorescence imaging.

### Statistics

Survival analysis of the mice was performed using Kaplan-Meier survival curves. For in vivo and ex vivo flow cytometry analyses, one-way analysis of variance (ANOVA) tests with Dunnet’s multiple-comparisons tests were applied. All analyses were carried out using GraphPad Prism version 10 software (GraphPad Software Inc.), and *P* < 0.05 values were considered statistically significant.

### Immunological evaluations

We harvested tumors, spleens, and tumor-draining lymph nodes from mice at week 35 and processed them into single-cell suspensions by mechanical disruption through 40-μm mesh filters. We used samples from four mice that had tumors and were available at the time of analysis. We analyzed the total numbers of cells by flow cytometry and counted them for each tissue (table S1). We counted cell numbers for *x* volume of blood, whole spleen, whole tumor-draining lymph node, and *x* percentage of tumor. Blood was also harvested at this time, and red blood cells were removed by hemolysis. We identified CD45+ cells as CD45+ and Viability Dye− and gated all immune cells from this population. We gated NK cells as CD3− NK1.1+. We gated T cells as CD3+ NK1.1− and then divided them into CD4+ CD8− or CD4− CD8+ to identify CD4 and CD8 T cells, respectively. We gated B cells as CD19+ MHCII+. We gated M1 Macs as MHCII+ CD19− F4/80+ CD11b+ CD206− CD11c+. We gated M2 Macs as MHCII+ CD19− F4/80+ CD11b+ CD206+ CD11c−. We gated DCs as MHCII+ CD19− F4/80− CD11c+. We gated monocytes/granulocytes as MHCII+ CD19− F4/80− CD11c− (fig. S1).

We stained single-cell suspensions with Zombie UV (BioLegend, 423107) for 30 min in PBS. Samples were then washed and stained with surface antibodies (see the list below) for 30 min in staining buffer (PBS + 1% bovine serum albumin + 0.1% sodium azide). Cells were fixed, permeabilized, and stained for transcription factor expression using the Transcription Factor Staining Buffer Set (eBioscience, 00-5523-00) following the manufacturer’s protocol. We analyzed data on a Cytek Aurora using FlowJo. The antibodies used are as follows: CD3-PECy5 (BioLegend, 100274), CD4-BV650 (BioLegend, 100545), CD8a-V500 (BD, 560776), CD11b-PECy7 (BD, 552850), CD11c-BUV737 (BD, 612796), CD19-AlexaFluor700 (BD, 557958), CD45.2-BV711 (BioLegend, 109847), CD206-AlexaFluor647 (BioLegend, 141712), FoxP3-AlexaFluor488 (BioLegend, 320011), F4/80-BUV661 (BD, 750643), GATA3-PE (Invitrogen, 12-9966-42), Ly6G-BV786 (BD, 740953), MHC II (I-A/I-E)-APCeFluor780 (Invitrogen, 47-5321-80), NK1.1-BUV395 (BD, 564144), and Tbet-PerCPCy5.5 (Invitrogen, 45-5825-82).
