# Supplementary material for: An immunocompetent, multifluorescent resource for real-time analysis of osteosarcoma progression, metastasis, and response to therapy
Source: Sci Adv. 2026 Oct 2;12(40):eaed6938. doi: 10.1126/sciadv.aed6938 (PMC13632736; doi:10.1126/sciadv.aed6938)
Supplement: Supplementary file 1 [file sciadv.aed6938_sm.pdf]

Science Advances
AAAS

Supplementary Materials for

An immunocompetent, multifluorescent resource for real-time analysis of osteosarcoma progression, metastasis, and response to therapy

Taiana C. Leite *et al.*

Corresponding author: Giuseppe Intini, gii5@pitt.edu

Sci. Adv. **12**, eaed6938 (2026)
DOI: 10.1126/sciadv.aed6938

This PDF file includes:

Fig. S1
Table S1

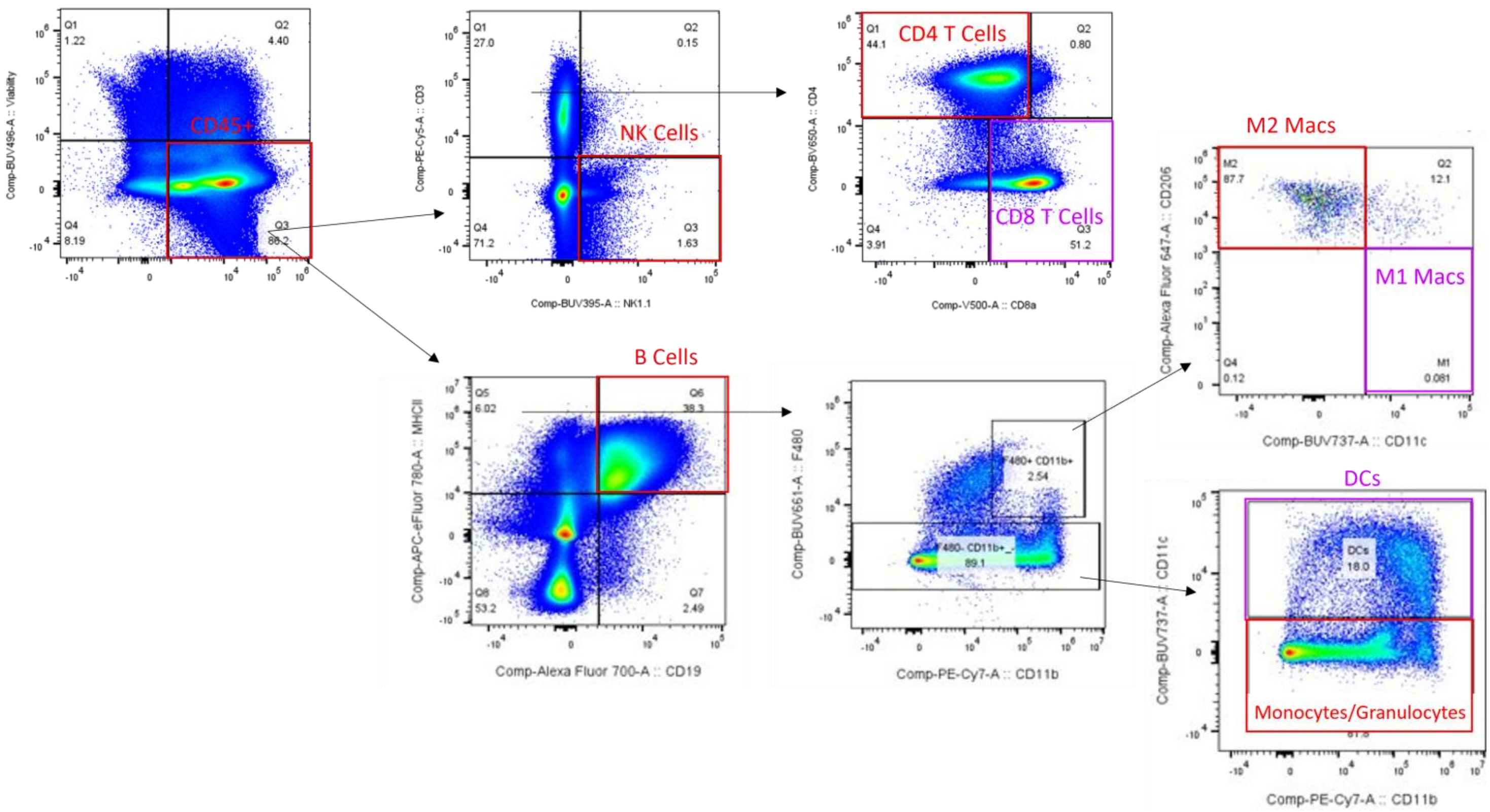

Supplemental Figure 1. Blood was also harvested at this time and RBCs removed by hemolysis. We identified CD45+ cells as CD45+ and Viability Dye- and gated all immune cells from this population. We gated NK cells as CD3- NK1.1+. We gated T cells as CD3+ NK1.1- and then divided them into CD4+ CD8- or CD4- CD8+ to identify CD4 and CD8 T cells respectively. We gated B cells as CD19+ MHCII+. We gated M1 Macs as MHCII+ CD19- F4/80+ CD11b+ CD206- CD11c+. We gated M2 Macs as MHCII+ CD19- F4/80+ CD11b+ CD206+ CD11c-. We gated DCs as MHCII+ CD19- F4/80- CD11c+. We gated Monocytes/Granulocytes as MHCII+ CD19- F4/80- CD11c-.

Mouse	Blood	Lymph Node	Spleen	Tumor
T75	5.8x10^5	9.00x10^5	1.22x10^6	1.76x10^5
T85	7.3x10^5	8.75x10^4	1.50x10^8	1.70x10^6
T84	4.2x10^6	1.04x10^6	2.92x10^8	5.23x10^5
T89	1.02x10^6	2.12x10^6	1.75x10^8	1.88x10^6

Supplemental Table 1. Total numbers of cells analyzed by flow cytometry for each tissue.
